# Hydrated electrons induce the formation of interstrand cross-links in DNA modified by cisplatin adducts

**DOI:** 10.1093/jrr/rraa014

**Published:** 2020-03-25

**Authors:** B Behmand, A M Noronha, C J Wilds, J-L Marignier, M Mostafavi, J R Wagner, D J Hunting, L Sanche

**Affiliations:** 1 Groupe en sciences des radiations, Faculté de médicine et des sciences de la santé, Université de Sherbrooke, Sherbrooke, Québec, J1H 5N4, Canada; 2 Department of Chemistry and Biochemistry, Concordia University, Montréal, Québec, H4B1R6, Canada; 3 Centre de cinétique rapide ELYSE, Laboratoire de chimie physique, Université de Paris-Saclay 11, Orsay, France

**Keywords:** radiotherapy, anticancer drugs, rate constant, pulse radiolysis, gel electrophoresis, oligonucleotide

## Abstract

Double-stranded oligonucleotides containing cisplatin adducts, with and without a mismatched region, were exposed to hydrated electrons generated by gamma-rays. Gel electrophoresis analysis demonstrates the formation of cisplatin-interstrand crosslinks from the cisplatin-intrastrand species. The rate constant per base for the reaction between hydrated electrons and the double-stranded oligonucleotides with and without cisplatin containing a mismatched region was determined by pulse radiolysis to be 7 × 10^9^ and 2 × 10^9^ M^−1^ s^−1^, respectively. These results provide a better understanding of the radiosensitizing effect of cisplatin adducts in hypoxic tumors and of the formation of interstrand crosslinks, which are difficult for cells to repair.

## INTRODUCTION

Platinum-based chemotherapeutic agents, including cisplatin (cisPt), are used for the treatment of several tumors, including ovary, lung, testicular, head and neck cancer [1–3], often in combination with radiotherapy, either sequentially or concomitantly (chemo-radiotherapy). The chemotherapeutic action of these agents occurs via DNA–cisPt adducts formed within the nucleus of cancer cells. The major DNA–cisPt adducts are intrastrand crosslinks which are readily repaired by nucleotide excision repair, with high fidelity. A very small amount of the total cisPt lesions are interstrand crosslinks (ICLs) [4], which are extremely toxic to cells, probably because they are difficult or impossible to repair in an error-free manner, depending on the phase of the cell cycle [5]. Ionizing radiation alone is not efficient at forming ICLs, however, Cecchini *et al*. have shown ICL formation by γ-rays exclusively in small mismatched regions of DNA containing a single bromouracil [6]. The position and efficiency of ICL formation was dependent on the nature of the mismatched sequences [7].

Following the interaction of ionizing radiation with cellular medium, low energy electrons (LEE) with energy <20 eV are produced in abundance. The presence of platinum adducts in DNA leads to radiosensitization of purified DNA, cultured cells and tumors. Although the precise mechanisms have not been completely elucidated, platinated DNA has been shown to be greatly sensitized to damage by LEE such that a single LEE can induce a double-strand break (DSB) [8] and LEE with energies near 0 eV can induce strand breaks [9].

In solution, the energetic electrons can react with solutes such as DNA/RNA bases [10–12], or can lose their energy via the collision with surrounding medium to become trapped and then hydrated. The hydrated electrons (e^−^_aq_) contribute very little to the toxicity of ionizing radiation because they do not create strand breaks or any complex form of DNA damage. Nevertheless, e^−^_aq_ are particularly interesting in the case of DNA modified by platinum adducts, for the following reasons: (i) e^−^_aq_ induce base damage near the sites of cisPt attachment and a single adduct can catalyze damage to several bases [13, 14]; (ii) the rate constant of the reaction between e^−^_aq_ and trinucleotide-cisPt is three times that of the trinucleotide without cisPt [15]; (iii) hydrated electrons are formed at high efficiency by ionizing radiation, with a yield equal to that of hydroxyl radicals with a G value of 0.28 μmol J^−1^ [16]; and (iv) hydrated electrons have a long half-life under hypoxia, and thus may offer a means of killing hypoxic tumor cells that are resistant to radiation, in part because oxygen is an excellent radiosensitizer [17, 18].

In the present work, the reaction of e^−^_aq_ with double-stranded oligonucleotide–cisPt complexes containing a mismatched region is investigated and the rate constant for the reaction of e^−^_aq_ with platinated DNA is determined by pulse radiolysis.

## MATERIALS AND METHODS

### Single-stranded oligonucleotides–cisPt complex

cisPt-modified oligonucleotides were prepared according to previously published protocols. To activate cisPt {conversion of *cis*-[Pt(NH_3_)_2_ Cl_2_] to *cis*-[Pt(H_2_O)(NH_3_)_2_Cl]^+^ or *cis*-[Pt(H_2_O)_2_(NH_3_)_2_]^2+^}, 4.5 mg of cisPt (15 nmol/μL) was treated with 12 μL of AgNO_3_ stock solution (2.5 μmol/μL) and diluted to 1 mL with 18 MΩ Millipore water. The samples were shaken vigorously in the dark at 37°C for 16 h. The silver chloride precipitate was removed by centrifugation and the activated cisPt (supernatant) was recovered. The single-stranded oligonucleotide sequences (100 nmol) were treated with activated cisPt (150 nmol, 20 μL) in a 1 mL reaction mixture containing 8.4 mmol/L sodium perchlorate. The reaction mixture was shaken vigorously in the dark at 37°C for a minimum of 16 h. The reaction was checked by ion-exchange (IEX) HPLC to monitor if there was complete conversion. The samples were desalted by Nap 5 (GE Healthcare Life Sciences™) columns and quantitated prior to purification by ion-exchange (IEX) high-performance liquid chromatography (HPLC). All cisPt-modified oligonucleotides were purified by IEX HPLC using a Dionex DNAPAC PA-100 column (0.4  × 25 cm, purchased from Dionex Corporation) using a linear gradient of NaCl from 0–25% of 1 M NaCl in 0.1 M Tris HCl at pH 7.6 and 10% acetonitrile for 10 min then 25–55% of 1 M NaCl in 0.1 M Tris HCl at pH 7.6 and 10% acetonitrile for the next 20 minutes at 21°C. The eluent was monitored at 260 nm for analytical runs or 280 nm for preparative runs. The purified oligomers were desalted using NAP 5 columns. All purified samples were dried on a Savant Speed-Vac® Concentrator and redissolved in water for electrospray ionization mass spectrometry (ESI-MS) analysis. The molecular mass of the cross-linked oligomer was determined by ESI-MS and agreed with the calculated value (7351 Da).

### Double-stranded oligonucleotides

The complementary and semi-complementary oligonucleotides 5′-GAG AGG AGA GAC ACA GAG AGA GGA-3′ (SS CAC) and 5′-GAG AGG AGA GTA TAT GAG AGA GGA-3′ (SS ATA) were purchased from the DNA synthesis laboratory at the University of Calgary, Alberta, Canada. The single-stranded oligonucleotides were 3′ end-labeled with γ-^32^P ddATP using terminal deoxynucleotidyl transferase (DTD) and buffer. Afterward, they were purified with a G-50 Sephadex microcolumn. The single-stranded SS GTG-cisPt and SS GTG were hybridized to complementary SS CAC and semi-complementary SS ATA with 2-fold excess of the unlabeled strand. These samples were heated to 82^°^C for 5 min and cooled down slowly for 3 h. To determine the hybridization of the oligonucleotides, non-denaturing polyacrylamide gel electrophoresis was used as described previously [19].

### Experimental conditions

The concentration of the oligonucleotides was 5 × 10^−5^ M. ^•^OH radicals were scavenged with 25 mM ethylenediaminetetraacetic acid (EDTA). Using the equation for competition kinetics [20],

(1) \begin{equation*} \frac{\mathrm{k}\left(^{\bullet} \mathrm{OH}\!+\!\mathrm{EDTA}\right)\!\times\! \left[\mathrm{EDTA}\right]}{\left(\mathrm{k}\left(^{\bullet} \mathrm{OH}\!+\!\mathrm{EDTA}\right)\!\times\! \left[\mathrm{EDTA}\right]\right)\!+\!\left(\mathrm{k}\left(^{\bullet} \mathrm{OH}\!+\!\mathrm{DNA}\right)\!\times\! \left[\mathrm{DNA}\right]\right)}\times 100 \end{equation*}

99.9% of ^•^OH radicals were eliminated by this concentration of scavenger.

The oligonucleotide solutions were bubbled with wet nitrogen gas (purity of 99.998%) for 3 min to minimize oxygen, which is a scavenger of hydrated electrons. The concentration of O_2_ in aqueous solutions under atmospheric conditions is 2.18 × 10^−4^ M. In our conditions after bubbling the solution with N_2_ it is expected to be reduced to ~20 μM in 3 min [21].

### Irradiation

Oligonucleotide solutions were irradiated in a ^137^Cs Gammacell with the dose varying between 1500 and 2000 Gy.

### Denaturing gel electrophoresis

The single- and double-stranded oligonucleotides were loaded on a 7 M urea denaturing 20% polyacrylamidegel.

### Pulse radiolysis

Transient absorption spectra from the electron pulses were recorded with the streak camera of the picosecond pulse radiolysis setup at the ELYSE facility located in Paris-Sud University. The facility delivered the electron beam irradiation with a repetition rate of 1 Hz. The dose of 10 Gy per pulse deposited in the samples is deduced from the measurements of the transient absorbance of e^−^_aq_ in water and verified before each experiment. The details of the system are described elsewhere [22, 23]. The sample, in an optical quartz cell with a length of 1 cm, is placed in experimental area EA-3 and bubbled with argon to eliminate oxygen. *Tert*-butanol with a concentration of 0.2 M was added to the solution to scavenge the ^•^OH and H^•^ radicals.

## RESULTS

### ICL formation

**Fig. 1.**
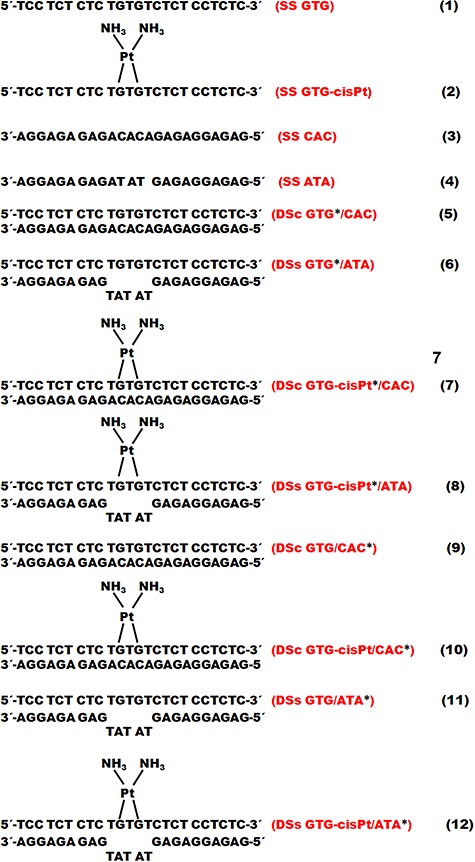
Sequences of oligonucleotides. The stranded oligonucleotides labeled with 32P are indicated by an asterisk. The hybridization is indicated as follows: single-stranded (SS), double-stranded complementary (DSc), and double-stranded semi-complementary (DSs).

**Fig. 2.**
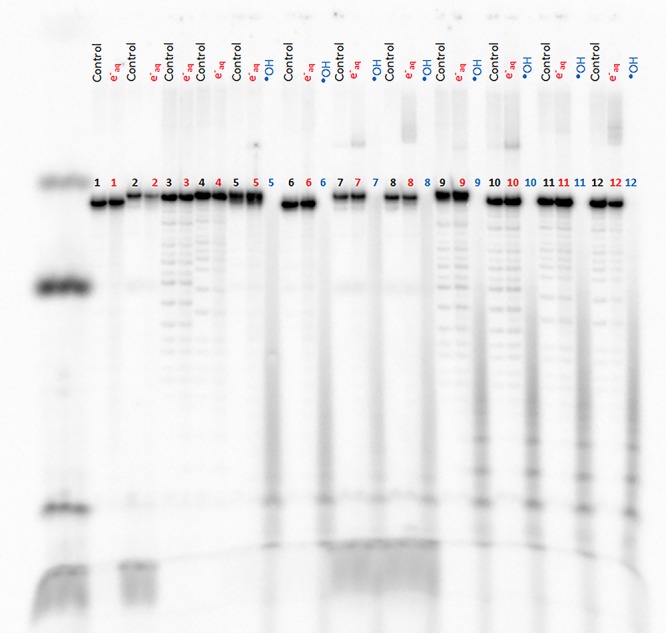
Denaturing polyacrylamide gel electrophoresis showing the effect of e^−^_aq_ and ^●^OH at a dose of 1500 Gy on SS GTG (lane 1), SS GTG-cisPt (lane 2), SS CAC (lane 3), SS ATA (lane 4), DSc GTG^*^/CAC (lane 5), DSs GTG^*^/ATA (lane 6), DSc GTG-cisPt^*^/CAC (lane 7), DSs GTG-cisPt^*^/ATA (lane 8), DSc GTG/CAC^*^ (lane 9), DSc GTG-cisPt/CAC^*^ (lane 10), DSs GTG/ATA^*^ (lane 11) and DSs GTG-cisPt/ATA^*^ (lane 12). Strands labeled with ^32^P are indicated by an asterisk.

The sequences of the different oligonucleotides investigated in this work are shown in Fig. 1. The interaction of the ^●^OH and e^−^_aq_ with the single- and double-stranded oligonucleotides with an irradiation dose of 1500 Gy are presented in Fig. 2.

The effect of e^−^_aq_ on the single-stranded oligonucleotides SS GTG, SS GTG–cisPt, SS CAC and SS ATA is shown in lanes 1–4, respectively. In contrast with previous observations [13–15], cisPt detachment by e^−^_aq_ was not detected for SS GTG–cisPt [2] by gel electrophoresis analysis. Here, the use of longer oligonucleotides compared to those in previous studies does not permit the separation between oligonucleotide and oligonucleotide–cisPt complex by the electrophoresis gel. The effect of e^−^_aq_ and ^●^OH on the double-stranded oligonucleotides, DSc GTG^*^/CAC, DSs GTG^*^/ATA, DSc GTG-cisPt^*^/CAC, DSs GTG-cisPt^*^/ATA, DSc GTG/CAC^*^, DSc GTG-cisPt/CAC^*^, DSs GTG/ATA^*^ and DSs GTG-cisPt/ATA^*^ are shown in lanes 5–12 respectively. The samples treated with ^●^OH produce a smear upon electrophoresis, indicating extensive degradation.

**Fig. 3.**
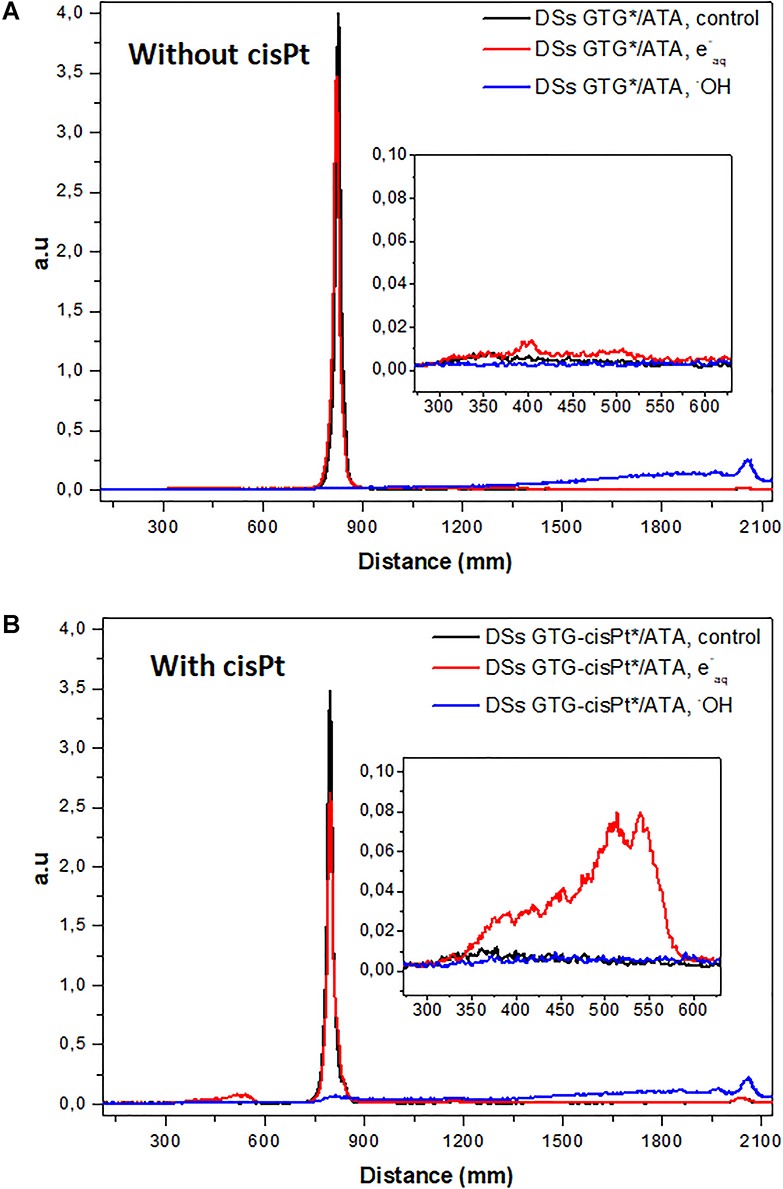
Graphical representation of lanes 11 and 12 in Fig. 2, showing ICL formation in a double-stranded oligonucleotide in arbitrary unity (a.u) with a mismatched region without and with cisPt adduct, as detected by denaturing gel electrophoresis. Insets show an enlargement for distances between 300 and 600 mm.

Fig. 3 shows the graphical representation of lanes 11 and 12, in which double-stranded oligonucleotides with a mismatched region, in the presence (b) or absence (a) of cisPt adducts, were exposed to ^●^OH or e^−^_aq_. As expected, ^●^OH damage created strand breaks for oligonucleotides with and without cisPt adducts, as shown by the loss of the parental peak located at 850 mm and the formation of an elongated smear extending from 1300 to 2100 mm. The red curve (Fig. 3) shows the e^−^_aq_ effect, where a new peak appears around 500 mm, but only in the presence of cisPt and only for double-stranded oligonucleotides. This slow-migrating peak is consistent with the formation of ICLs (lanes 7, 8, 10 and 12 in Fig. 2). No single-strand breaks were observed with e^−^_aq_, with or without cisPt, but this may be a question of sensitivity of detection (see Discussion).

The percentage of ICL formation for different doses was calculated by deconvolution of this broad peak and by using the Lorentzian–Gaussian fit (Table 1). This peak is not present for the single-stranded oligonucleotides.

**Table 1:** Percentage of ICL formation in double-stranded oligonucleotides for different radiation doses. Strands labeled with ^32^P are indicated by an asterisk

	ICL % 0 Gy	ICL % 1500 Gy	ICL % 2000 Gy
DSc GTG^*^/CAC	0.05	0.7	0
DSc GTG-cis Pt^*^/CAC	1. 07	4.1	7
DSc GTG/CAC^*^	0. 9	1. 6	0
DSc GTG-cis Pt/CAC^*^	1. 5	5.55	6
DSs GTG^*^/ATA	1.1	1.1	0
DSs GTG-cis Pt^*^/ATA	1.8	13 .5	14 .4
DSs GTG/ATA^*^	1. 35	1. 6	0
DSs GTG-cis Pt/ATA^*^	2.1	10	12.5

**Fig. 4.**
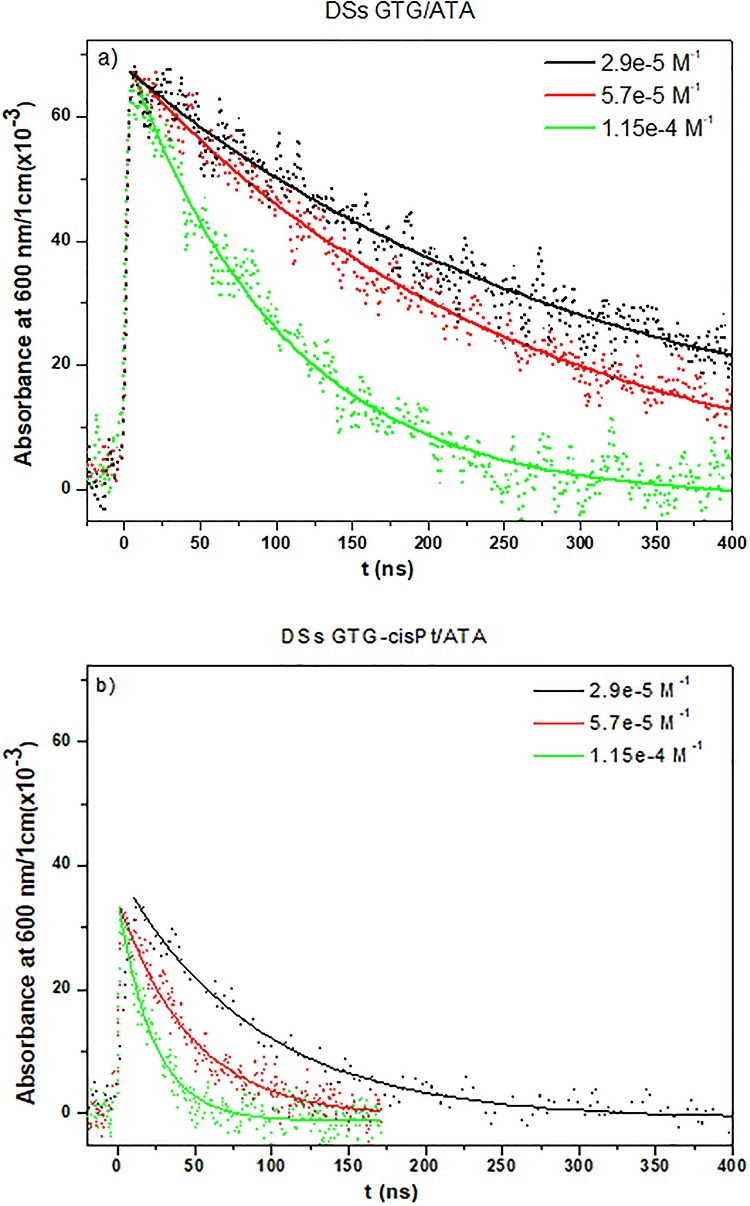
Nanosecond decay kinetics of e^−^_aq_ at 600 nm in (**a**) DSs GTG/ATA and (**b**) DSs GTG-cisPt/ATA solutions at different concentrations. The solutions were bubbled with Ar and contained *tert*-butanol.

**Fig. 5.**
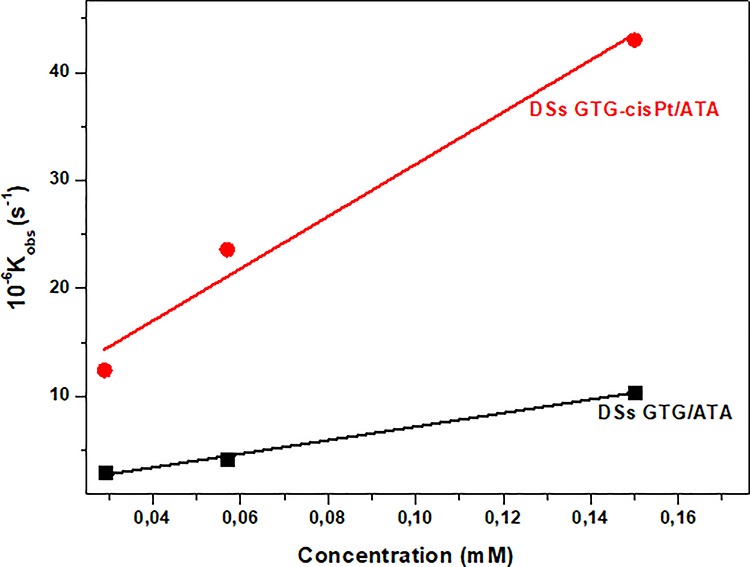
Pseudo-first-order rate constant *k*_obs_ vs DSs GTG-cisPt/ATA and DSs GTG/ATA.

In the absence of cisPt, there is no ICL formation in double-stranded DNA with or without a mismatched region. In the presence of cisPt, ICLs are formed with approximately the same yield whether the cisPt strand or the complementary strand is labeled with ^32^P. This is an important confirmation of the formation of ICLs between the complementary DNA strands. The formation of ICLs for DSs or DSc with a cisPt adduct is 2-fold more efficient in the case of oligonucleotides with a mismatched region compared to the corresponding oligonucleotides without a mismatched region. There is not much difference between 1500 and 2000 Gy; ICL seems to increase only slightly. Furthermore, we can assume that the effect of dose is linear considering the low conversions of the parent. The statistical variations in the measurements are not given as the number of repetitions of the experiments is too small. However, the variation of measured ICL is <2% for different doses.

### Reaction rates measured by pulse radiolysis

The rate of the reaction between e^−^_aq_ and DNA with a mismatched region (DSs GTG/ATA) with or without a cisPt adduct was determined by pulse radiolysis. Around 10 transient spectra were recorded and averaged for each solution irradiated with a dose of 10 Gy. The decay of e^−^_aq_ at 600 nm for DSs GTG/ATA and DSs GTG-cisPt/ATA is shown in Fig. 4a and b for different concentrations. The rate of decay is accelerated by the presence of a cisPt adduct.

The rate constants of the reactions were determined by a pseudo-first-order approximation because of the very low concentration per pulse of e^−^_aq_ (~3 × 10^−6^ M^−1^ at 3 ns). The decay of e^−^_aq_ on a logarithmic scale for each solution at different concentrations gives rise to the observed rate constant *k*_obs_ (Fig. 5). The rate constant for the mismatched oligonucleotide with a cisPt adduct is 3.5-fold higher than for the same oligonucleotide without cisPt (Table 2). The systematic error is ~10%.

## DISCUSSION

Previous water-radiolysis studies have shown that the presence of cisPt adducts sensitizes single-stranded oligonucleotides to e^−^_aq_, leading to the formation of base damage and loss of the platinum [13, 14]. Interestingly, the platinum appears to act as a catalyst, reacting with several electrons and generating, on average, 3.5 damaged bases before being lost. In the present study, we demonstrate that, in the presence of cisPt adducts, e^−^_aq_ produces ICL in double-stranded DNA. This is a surprising result with potential importance for chemoradiation therapy with platinum-based agents. Cancer cells in hypoxic regions of tumors are resistant to radiation; however, our finding that e^−^_aq_ induce crosslinks in platinated DNA suggest a strategy for attacking hypoxic cancer cells, since low oxygen concentrations will prolong the half-life of e^−^_aq_. The rate constant for the reaction of oxygen with e^−^_aq_ is 1.9 × 10^10^ M^−1^ s^−1^ [20] and therefore the e^−^_aq_ concentration will be reduced in normoxic regions of the tumor. ICLs are very toxic to all cells and particularly toxic to certain cancer cells lacking the full complement of repair pathways.

Our results showing no induction of DNA strand breaks by e^−^_aq_ in platinated oligonucleotides would seem to contradict previous results published by our group in which both single- and double-strand breaks were observed [8]. However, this apparent incoherence is almost certainly the result of the large differences in the sensitivity of detection of strand breaks. Our previous study used supercoiled plasmid DNA. A unique single-strand break anywhere in the 3400 bp plasmid generates a relaxed plasmid with a different migration rate during gel electrophoresis, giving rise to a single band. This offers much higher sensitivity than a 24 bp oligonucleotide.

The average rate constant per base (48 bases) in the oligonucleotide containing a mismatched region is found to be 2 × 10^9^ and 7 × 10^9^ M^−1^ s^−1^ in the absence and presence of cisPt, respectively. These rate constants agree with the measurements performed in shorter single-stranded oligonucleotides [15].

The results show that the presence of only one cisPt adduct per 48 nucleotides makes double-stranded oligonucleotide 3.5 times more reactive than double-stranded oligonucleotide alone. The formation of transient anions by attachment of near-zero-electrons to the bases may occur in water [24]. Our results show that the presence of Pt, which has high electron affinity, decreases the energy barrier of trapped hydrated electrons, such that dissociative electron transfer becomes the mechanism responsible for the rupture of one cisPt–guanine bond followed by formation of ICL.

Initially, an e^−^_aq_ can add to the Pt–DNA complex to form the corresponding anion radical. The latter anion radical may either undergo a transfer or a bond-cleavage reaction. In the case of transfer, the electron may undergo transfer to a DNA base to give a DNA base anion radical. Electron transfer to T will be the most probable because this base has the highest electron affinity. In turn, T radical anions can transform into a T modification, e.g., 5,6-dihydrothymine, which may explain the loss of thymine in previous studies with Pt–DNA using single-stranded oligonucleotides [14]. In the case of a bond-cleavage reaction, the anion radical of the Pt–DNA complex may undergo cleavage of the Pt–G bond leading to a reactive Pt^I^ species, which in turn, reacts with the opposite strand to produce an ICL. The presence of a large number of purine sites in close vicinity to the cisPt favors a reaction between Pt^I^ and a purine base. Alternatively, the bond-cleavage reaction may produce a reactive G species that is responsible for ICL formation. Cleavage of the Pt–G bond has previously been observed by mass spectrometry in the negative mode with an oxaliplatin–DNA intrastrand cross-link [25]. In addition, irradiation of oligonucleotides containing Pt also results in the loss of G due to the reactions of hydrated electrons [14]. Further studies are in progress to identify the main products of the reaction of hydrated electrons with Pt–DNA complexes.

Finally, our results show that the presence of a mismatched region increases the yield of ICL. This is consistent with the idea that conformation is crucial for ICL formation. It has been shown that the reactivity of mismatched regions is dependent on the nature of the bases surrounding the initial radical [7]. In addition, mismatched regions have been shown to disturb charge transfer along the double helix [26, 27], which could make the electron transfer to cisPt more favorable. The subsequent rupture of one cisPt–G would be followed by either the loss of the Pt or by cisPt binding to guanine or adenine of the opposite strand.

**Table 2:** Rate constant of the e^−^_aq_ reaction with DSs GTG- cisPt/ATA and DSs GTG/ATA

Sample	Rate constant/base (M^−1^ s^−1^)
DSd GTG/ATA	2 x10^-9^
DSd GTG-cisPt/ATA	7 x10^-9^

In conclusion, interaction of e^−^_aq_ with double-stranded oligonucleotide–cisPt adducts shows the formation of ICL in the presence and absence of a mismatched region. The detachment of the cisPt adduct from single-stranded oligonucleotides exposed to hydrated electrons has been observed previously [13–15]. In the present study, the dissociation of one cisPt–guanine bond due to the high electron affinity of cisPt, which attracts e^−^_aq_ to the cisPt site, could explain the formation of ICL, following the formation of reactive Pt^I^. ICL formation is 2-fold higher in oligonucleotides containing mismatched regions compared to oligonucleotides without mismatched regions. The pulse radiolysis experiment demonstrates that a mismatched double-stranded oligonucleotide–cisPt complex is 3-fold more reactive than a mismatched double-stranded oligonucleotide without cisPt. These results reveal the probable mechanism of radiosensitization of double-stranded DNA by cisPt under hypoxic conditions.
